# Complete genome sequence of the kiwifruit bacterial canker pathogen *Pseudomonas savastanoi* strain MHT1

**DOI:** 10.1186/s12866-022-02459-4

**Published:** 2022-02-04

**Authors:** Mingzhao Zhong, Yunhao Sun, Xianzhi Zhang, Hong Liang, Lina Xiong, Qunxin Han

**Affiliations:** 1 Innovative Institute for Plant Health, Zhongkai University of Agriculture and Engineering, Guangzhou, China; 2 College of Resources and Environment, Zhongkai University of Agriculture and Engineering, Guangzhou, China; 3 College of Agriculture and Biology, Zhongkai University of Agriculture and Engineering, Guangzhou, China; 4 Guangdong Zhongkai Technology Development Co., Ltd, Huizhou, China; 5 School of Life Sciences, Sun Yat-sen University, Guangzhou, China; 6 Present Address: Guangzhou, People’s Republic of China

**Keywords:** *Pseudomonas savastanoi*, Kiwifruit bacterial canker, Comparative genomics, Effector, Genome

## Abstract

**Background:**

*Pseudomonas savastanoi* is an important plant pathogen that infects and causes symptoms in a variety of economically important crops, causing considerable loss of yield and quality. Because there has been no research reported to date on bacterial canker of kiwifruit (*Actinidia chinensis*) plants caused by *P. savastanoi* and, in particular, no in-depth studies of the complete genome sequence or pathogenic mechanism, long-lasting and environmentally friendly control measures against this pathogen in kiwifruit are lacking. This study therefore has both theoretical value and practical significance.

**Results:**

We report the complete genome sequence of *P. savastanoi* strain MHT1, which was first reported as the pathogen causing bacterial canker in kiwifruit plants. The genome consists of a 6.00-Mb chromosome with 58.5% GC content and 5008 predicted genes. Comparative genome analysis of four sequenced genomes of representative *P. savastanoi* strains revealed that 230 genes are unique to the MHT1 strain and that these genes are enriched in antibiotic metabolic processes and metabolic pathways, which may be associated with the drug resistance and host range observed in this strain. MHT1 showed high syntenic relationships with different *P. savastanoi* strains. Furthermore, MHT1 has eight conserved effectors that are highly homologous to effectors from *P. syringae*, *Pseudomonas amygdali*, and *Ralstonia solanacearum* strains. The MHT1 genome contains six genomic islands and two prophage sequences. In addition, 380 genes were annotated as antibiotic resistance genes and another 734 as encoding carbohydrate-active enzymes.

**Conclusion:**

The whole-genome sequence of this kiwifruit bacterial canker pathogen extends our knowledge of the *P. savastanoi* genome, sets the stage for further studies of the interaction between kiwifruit and *P. savastanoi*, and provides an important theoretical foundation for the prevention and control of bacterial canker.

**Supplementary Information:**

The online version contains supplementary material available at 10.1186/s12866-022-02459-4.

## Introduction

Bacterial canker of kiwifruit (*Actinidia* spp.) is a serious threat to the kiwifruit industry that causes substantial crop losses worldwide [1]. After the first symptoms of canker disease (such as leaf spots) appear, the disease spreads quickly to the rest of the plant, which can be destroyed within 1 year [2]. The main methods of disease control and mitigation include chemical treatment, orchard management, and breeding of resistant varietie, however, there are currently no effective methods to directly kill the causal pathogen [2]. Previous studies have reported that *P. syringae* pv. *actinidiae* is one of the main pathogenic agents of *Actinidia* spp. pathogenic bacterial canker disease [1–6].

*P. syringae* is an important phytopathogenic bacterium and one of the most relevant models for research on microbe–host interactions [7]. *P. savastanoi* is part of the *P. syringae* complex, which encompasses more than 60 pathovars, and *P. savastanoi* is recognized as a later synonyms of *P. amygdali* [8]. It has been reported that *P. savastanoi* could causes various diseases are on different host plants. *P. savastanoi* pv. *savastanoi* is usually reported as the causal agent of olive (*Olea europaea*) knot disease, which manifests during rainy months with moderate temperatures (10–20 °C) [9]; *P. savastanoi* pv. *fraxini* causes cankers accompanied by excrescences in European ash (*Fraxinus excelsior*) [10]; *P. savastanoi* pv. *nerii* induces knots in oleander (*Nerium oleander*), olive and ash [9]; *P. savastanoi* pv. *retacarpa* induces knots in broom (*Retama sphaerocarpa*) [9]; *Pseudomonas savastanoi* pv. *mandevillae* pv. nov., a clonal pathogen causing an emerging, devastating disease of the Ornamental plant *Mandevilla* spp. [11].

A growing number of *P. savastanoi* strains have been isolated and their genomes sequenced. Indeed, the National Center for Biotechnology Information (NCBI) database (https://www.ncbi.nlm.nih.gov/genome/) contains nearly 130 reports of genome assembly and annotation for *P. savastanoi* strains. This increasing amount of information highlights the biodiversity of *P. savastanoi* strains and has also helped facilitate a growing understanding of the pathogen’s underlying pathogenic mechanisms, gene regulatory networks, and evolutionary processes [9, 12, 13]. However, no genome sequence of a *P. savastanoi* strain that causes bacterial canker in *Actinidia* spp. plants is currently available, underscoring the need for a more comprehensive and detailed comparative genomics analysis of *P. savastanoi*.

In this work, we sequenced the genome of *P. savastanoi* strain MHT1, which was isolated from an infected golden kiwifruit (*Actinidia chinensis*) plant. Further, by comparative evolutionary and genomics analyses with other sequenced *P. savastanoi* strains, we investigated host-specific candidate genes, conserved type III secretion system effector proteins, and their evolutionary relationships.

## Results

### Isolation of pathogenic bacteria and pathogenicity tests

**Fig. 1:**
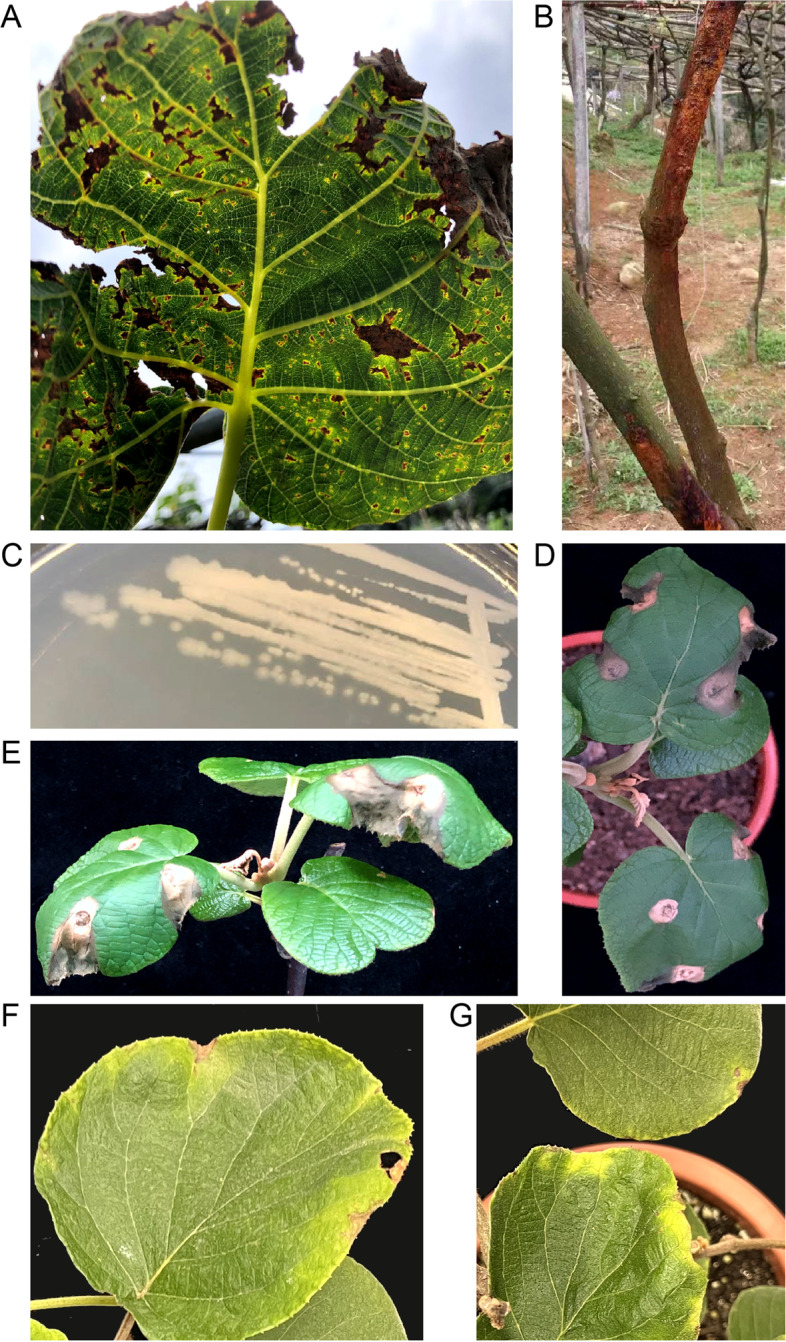
Isolation and pathogenicity tests of *Pseudomonas savastanoi* strain MHT1. **A** and **B**. Representative leaf and stem of *Actinidia chinensis* plants with bacterial canker symptoms sampled from the Xiache kiwifruit plant base (115°04′N, 24°64′E), Heping County, Heyuan City, Guangdong Province, China. Morphology of *Pseudomonas savastanoi* MHT1 colonies. A single colony of the MH1 strain was streaked and cultivated on LB medium at 28 °C for 24 h. **D** and **E**. Symptoms on the leaves and tender buds of *A. chinensis* plants (HongYang kiwifruit in **C**; WuZhi kiwifruit in **D**) generated by *P. savastanoi* MHT1 strains 8 days post-inoculation. *A. chinensis* leaves were infiltrated with bacterial suspensions (10^8^ CFU/mL in 1 mM MgCl_2_) and photographed 8 days later. CFU, colony-forming units. Negative control, leaves infiltrated with 10 mM MgCl_2_. **F** and **G**. Symptoms on the leaves of *A. chinensis* plants (HongYang kiwifruit) generated by *P. savastanoi* MHT1 strains 6 days post-infection. Abaxial side leaves of *A. chinensis* were sprayed with bacterial suspensions (10^8^ CFU/mL in 1 mM MgCl_2_) and photographed 6 days later

Bacterial canker disease was observed in the kiwifruit cultivars ‘HongYang’ and ‘WuZhi’ at the Xiache kiwifruit plant base (115°04′N, 24°64′E) in Heping County, Heyuan City, Guangdong Province, China. Symptoms such as cankers, cracks, and lesions with halos appeared on the leaves and trunks during early spring (Fig. 1A and B) under climatic conditions consisting of low temperatures, strong winds, and heavy rainfall. During year of 2019–2021, samples of disease leaves were collected the at three different time points. From the different samples, we isolated one same bacterium forming white colonies on plates (Fig. 1C), which was identified as pseudomonads by phylogenetic analysis by 16S rDNA and *SyrB* gene [14] (Figure S1). By The isolated strain reproduced typical symptoms on the leaves and tender buds of HongYang and WuZhi kiwifruit plants when infiltrated into the leaves (Fig. 1D and E) or spraying onto the abaxial sides of the leaves (Fig. 1F and G). We named this pathogenic strain MHT1, which is an acronym for the Chinese pronunciation of kiwifruit (Mi Hou Tao).

### Genome sequencing, assembly, and functional annotation

**Fig. 2:**
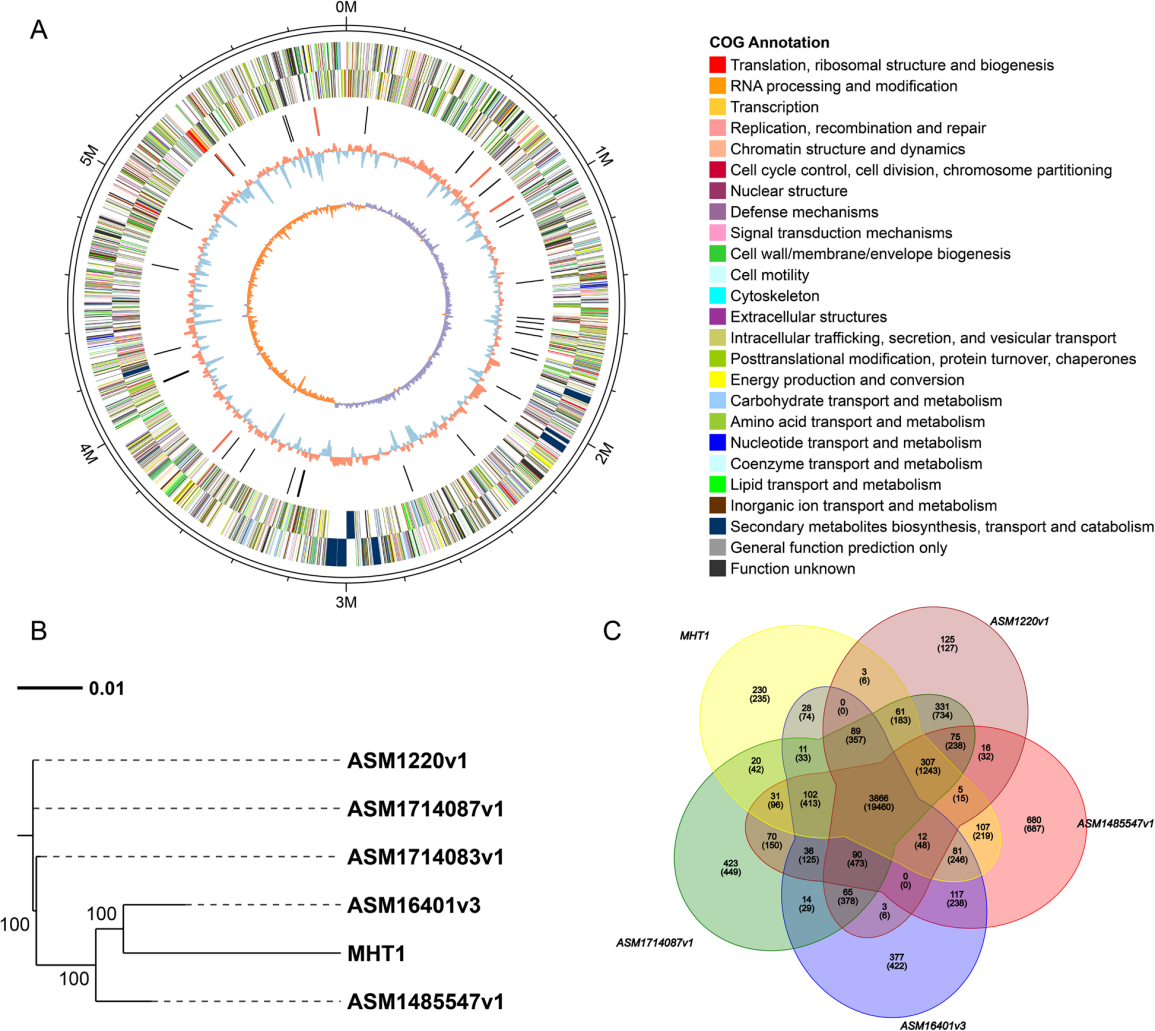
General genomic features of *Pseudomonas savastanoi* MHT1 strain. Circos plot of the MHT1 chromosome. The MHT1 chromosome contig has a full length of 5,999,881 bp and a GC content of 58.5% and harbors 5008 predicted genes. Innermost circle, GC skew curve (purple and orange indicating a GC skew lower or higher than zero, respectively); second circle out, GC content (blue and orange indicate GC content lower or higher than the average GC content across the genome, respectively); third circle, non-coding RNAs, with tRNAs in black and rRNA in red; fourth and fifth circles, annotated genes on the positive and negative strands, respectively, with colors representing the different classifications of COG functions; sixth (outermost) circle, size of the chromosome. **B**. Phylogenetic tree based on the whole genomes of five representative *P. savastanoi* strains and the MHT1 strain. **C**. Venn diagram showing the extent of overlap between gene families between MHT1 and the four representative *P. savastanoi* strains

To obtain more information about this pathogen and to explore the interaction mechanisms of the MHT1 strain with kiwifruit, we sequenced the MHT1 genome using the PacBio Sequel platform (Genedenovo Biotechnology Co., Ltd., Guangzhou, China). We obtained 1.12 Gb of reads representing an 187-fold coverage of the entire genome by SMRT sequencing (Genedenovo Biotechnology Co., Ltd., Guangzhou, China). After removing ambiguous and low-quality reads and adapters, we assembled the clean data into a single molecule of approximately 6.00 Mb (Fig. 2A) with the program MECAT [15]. The circular chromosome harbored 5008 predicted genes and exhibited a GC content of 58.5% (Table 1). We used several complementary strategies to predict the number of different RNAs, clustered regularly interspaced short palindromic repeats (CRISPR), genomic islands (GIs), prophage sequences, interspersed repeats, tandem repeats, and transposons along the chromosome (Table 1. The distribution statistics of the MHT1 gene complement against the Non-Redundant Protein Database (NR) (NCBI) showed that strain MHT1 shares 1949 and 1186 genes with *P. syringae* and *P. savastanoi*, respectively (Figure S2 and Table S1). According to the observed average nucleotide identity (ANI) (Figure S3), we refer to this strain as *Pseudomonas savastanoi*.

**Table 1:** General feature of the *Pseudomonas savastanoi* strain MHT1 genome

Features	Total
Size (bp)	5,999,881
G + C content (%)	58.47
Coding genes	5008
tRNA	63
23S_rRNA	5
16S_rRNA	5
5S_rRNA	6
sRNA	19
CRISPR number	6
Genomic islands	4
Prophage	2
Interspersed repeats	82
Tandem repeats	204
Transposon	2

### Genetic relationship between MHT1 and other *Pseudomonas savastanoi* strains

**Fig. 3:**
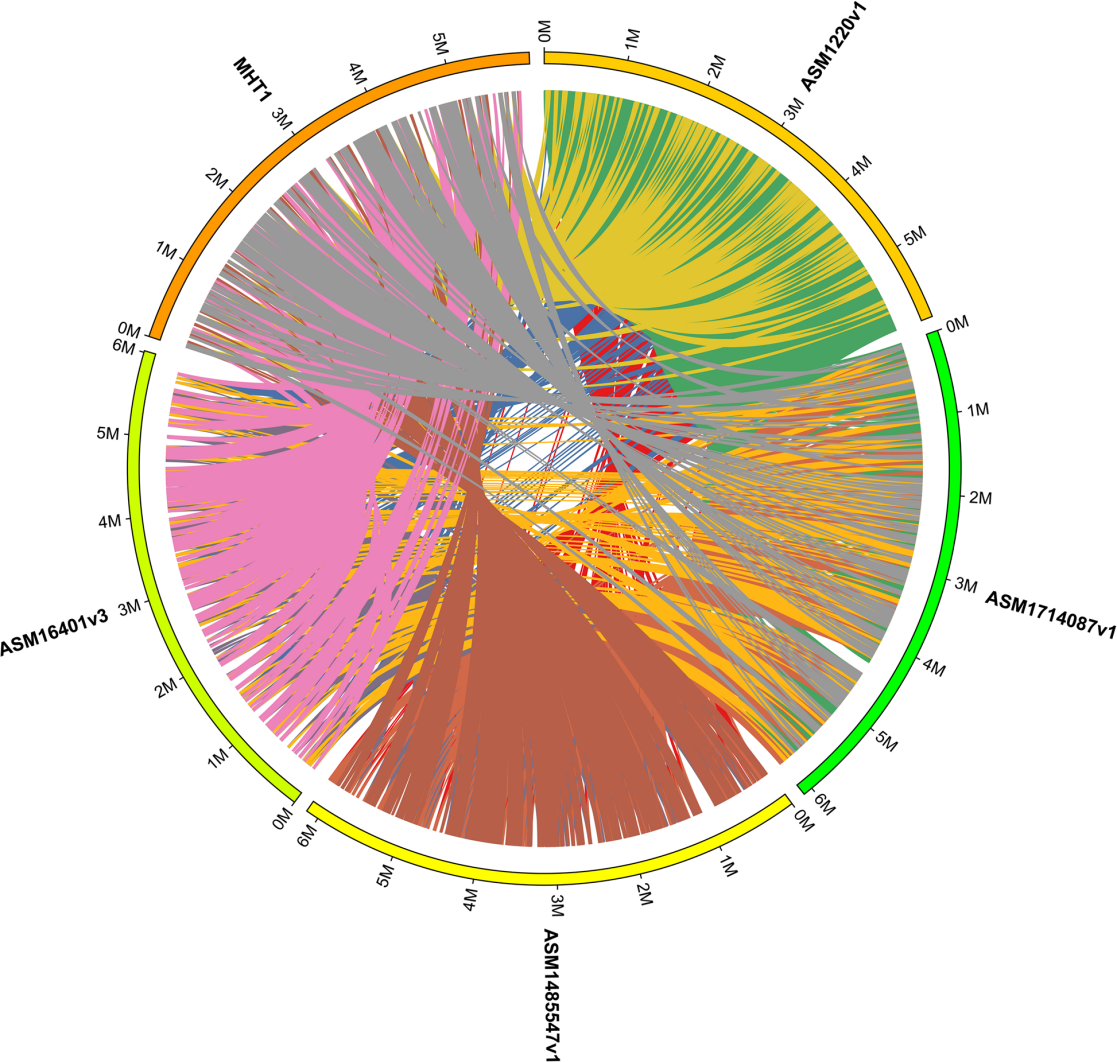
Synteny map of the genomes of MHT1 and four representative *P. savastanoi* strains: ASM1220v1, ASM1714087v1, ASM1485547v1, and ASM16401v3

We performed a phylogenetic analysis between MHT1 and four representative sequenced strains of *P. savastanoi* that have not been reported to cause bacterial canker on kiwifruit (assemblies ASM1220v1, ASM16401v3, ASM1485547v1, and ASM1714087v1)(Table S2) based on their whole genomes using the REALPHY platform [16] (Fig. 2B). We also compared these five *P. savastanoi* strains and investigated their strain-specific and shared genes (Fig. 2C). We determined that 230 genes are unique to MHT1. We identified the extent of synteny between MHT1 and the four representative strains using the C-Sibelia program [17] (Fig. 3). Collinear blocks in the MHT1 genome accounted for 87.8% (versus ASM1485547v1), 87.2% (versus ASM1220v1), 84.7% (versus ASM1714087v1), and 82.8% (versus ASM16401v3) of the total genome (Table 2).

**Table 2:** Comparison of collinearity between MHT1 and other strains of *Pseudomonas savastanoi*

Strain	Collinear with SY1 (%)
ASM1485547v1	87.82
ASM1220v1	87.24
ASM1714087v1	84.70
ASM16401v3	82.82

**Fig. 4:**
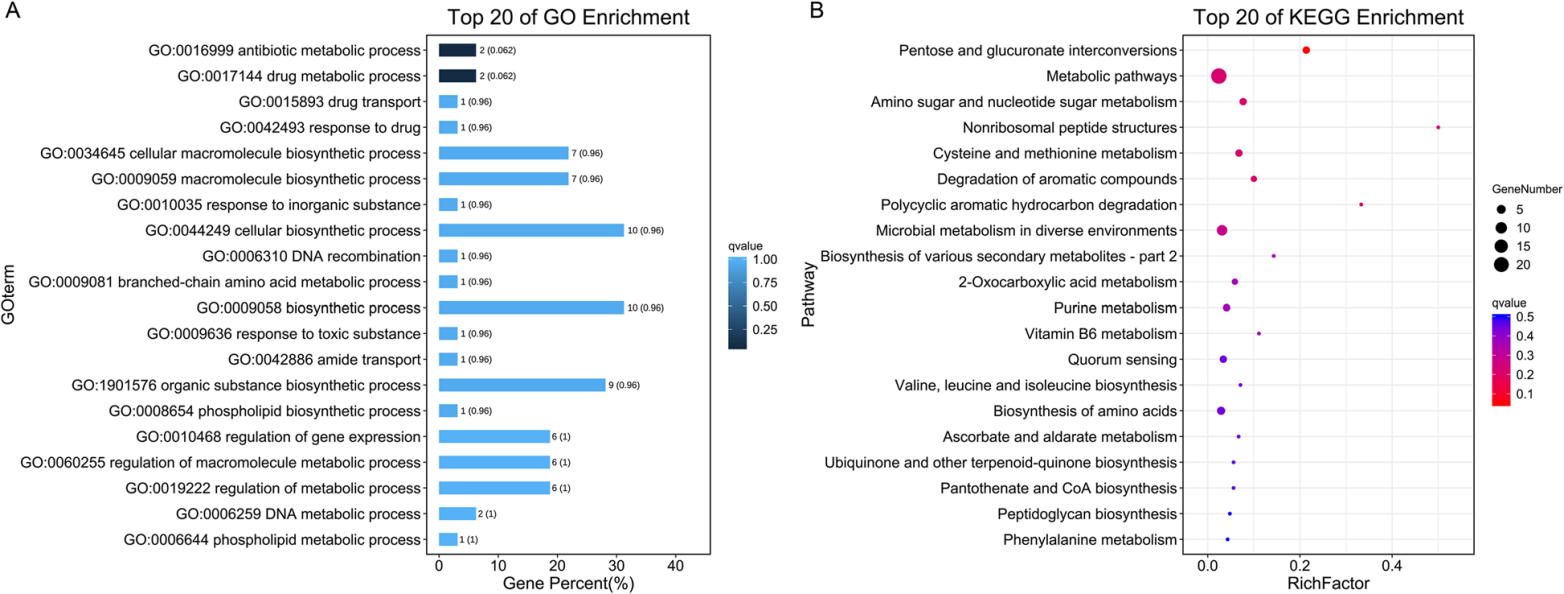
Top 20 enriched GO and KEGG pathways for MHT1-specific genes compared to the ASM1220v1, ASM1714087v1, ASM1485547v1, and ASM16401v3 strains. **A**, Enriched GO categories. **B**, Enriched KEGG pathways

We performed Gene Ontology (GO) [18] enrichment analysis to evaluate the possible function of these MHT1-specific genes: We observed enrichment for the GO terms ‘antibiotic metabolic process’ and ‘drug metabolic process’ (Fig. 4A). Subsequently, we performed a Kyoto Encyclopedia of Genes and Genomes (KEGG) [19] pathway analysis with the same MHT1-specific genes. The top enriched KEGG pathways were associated with ‘pentose and glucuronate interconversions’, ‘metabolic pathways’, ‘amino sugar and nucleotide sugar metabolism’, ‘nonribosomal peptide structures’, ‘cysteine and methionine metabolism’, and ‘degradation of aromatic compounds’ (Fig. 4B). These results showed that although MHT1 has high similarity with these four *P. savastanoi* strains, it still has many unique genes that are enriched in several physiological pathways.

### Prediction and analysis of conserved type III effectors in *Pseudomonas savastanoi* strain MHT1

Like many other gram-negative plant pathogenic bacteria, *P. savastanoi* may delivers many different type III effector proteins (T3Es) into host plants for its pathogenesis via a type III secretion system (T3SS) [13]. Given that different pathovars of *P. savastanoi* can infect several plant hosts, each strain may carry an array of conserved effector proteins that largely determines their host range [13]. To explore the characteristics of the MHT1 conserved effector protein arsenal, we searched for potential type III effectors encoded by the MHT1 genome using the T3E database and the program EffectiveT3 [20, 21]. We thus identified 26 proteins related to the T3SS and T3Es in the MHT1 genome (Table S3). We next looked for these T3Es in other *P. syringae*, *P. amygdali*, and *Ralstonia solanacearum* strains whose genomes have been sequenced and that can infect plant hosts [20]. We obtained eight conserved T3Es in MHT1 with strong similarity to the HopM1, AvrE1, and HopA1 effectors of *P. syringae* strains; the HopAH1and HopI1 effectors of *P. amygdali* strains; and the RipQ, RipW, and RipBG effectors of *R. solanacearum* strains (Table 3) [22–27]. We also predicted the subcellular localization of these potential effectors using Plant-mPLoc (http://www.csbio.sjtu.edu.cn/bioinf/plant-multi/) and Protein Homology/analogY Recognition Engine V 2.0 (http://www.sbg.bio.ic.ac.uk/phyre2/html/page.cgi?id=index) (Table 3). These results suggest that the existence of several conserved effector proteins may be important to the pathogenicity of MHT1.

**Table 3:** Predicted type III effectors of MHT1

Name	geneID in MHT1	Representative strain contains the homologous protein	Length (aa)	AA identity (%)	Predicted subcellular localization	Predicted functional domain	Description
HopM1	KP808_05995	*Pseudomonas syringae* pv. *Lachrymans*(MAFF301315)	744	97.0	Nucleus	Maguk p55 subfamily member 7	HopM1 effector of the bacterial pathogen *Pseudomonas syringae* pv. *tomato*, which destabilizes MIN7 leading to its degradation and weakening host defenses [22].
AvrE1	KP808_06005	*Pseudomonas syringae* pv. *Lachrymans*(MAFF301315)	1571	98.6	Cytoplasm	two-component system sensor histidine	Required for *in planta* growth and lesion production [23].
HopA1	KP808_06050	*Pseudomonas syringae* pv. *Lachrymans*(MAFF301315)	281	99.6	Chloroplast	Pyruvate dehydrogenase-dihydrolipoyl acetyltransferase	Required for effector-triggered immunity [24, 25].
HopAH1	KP808_06060	*Pseudomonas amygdali* pv. *tabaci* (ATCC 11528)	416	93.5	Cytoplasm	endoglucanase	Not identified
HopI1	KP808_06065	*Pseudomonas amygdali* pv. *aesculi* (NCPPB 3681)	232	95.3	Endoplasmic reticulum/ Nucleus.	J-domain of dnaj homolog dnj-2	Chloroplast Hsp70 is targeted by HopI1 to promote bacterial virulence by suppressing plant defenses [26].
RipQ	KP808_06095	*Ralstonia solanacearum* (GMI1000)	495	73.3	Chloroplast/ Cytoplasm/ Nucleus	S-adenosylmethionine methyltransferase	Inhibition of flg22-induced reactive oxygen species production in *N. benthamiana* [27].
RipW	KP808_11300	*Ralstonia solanacearum* (GMI1000)	426	61.6	Cell membrane/ Nucleus	endo-pectate lyase	Not identified
RipBG	KP808_11320	*Ralstonia solanacearum* (Molk2)	544	84.5	Nucleus	kinaseinteracting domain	Not identified

### Genomic islands and prophage elements

**Fig. 5:**
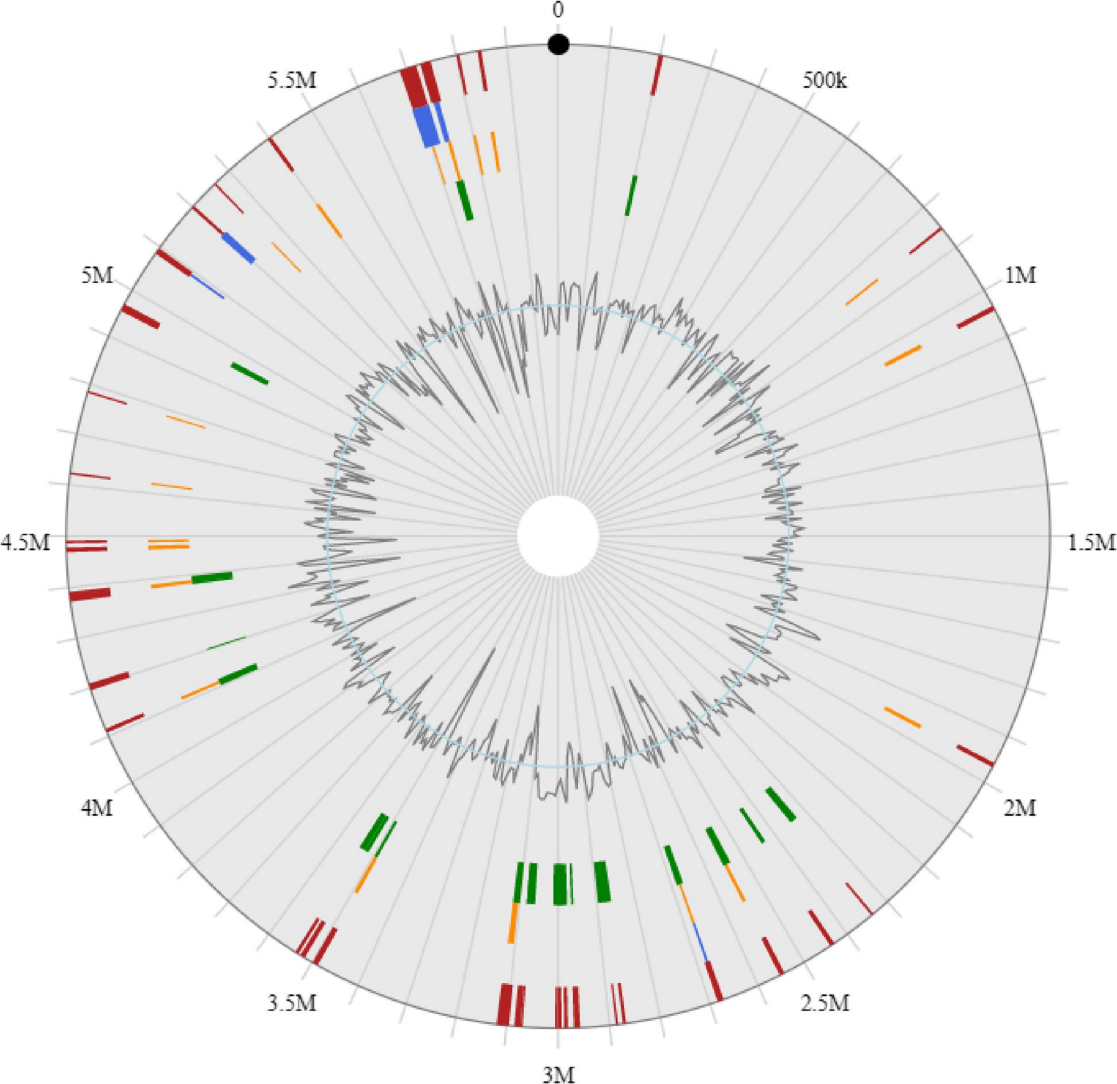
Circular and horizontal plots of genomic islands (GIs) identified along the MHT1 chromosome. Color bars represent the predicted GIs identified by SIGI-HMM (orange), IslandPick (green), and IslandPath-DIMOB (blue) as well as integrated results (red) from innermost to outermost circles

Genomic islands (GIs) are fragments of DNA derived from horizontal gene transfer between different bacterial genomes [28]. We screened the genome of MHT1 for GIs using Island viewer software [29]. We identified six GIs in the genome (Fig. 5) with the IslandPath-DIMOB program [29]. Over the entire chromosome, the lengths of GIs ranged from 12,298 to 64,840 bp, with an average size of 26,497 bp and covering a total length of 105,988 bp. These GIs contained 107 genes (Table S4), which mainly encoded FAD-binding oxidoreductases, short-chain dehydrogenase/reductase (SDR) family oxidoreductases, NAD(P)-dependent alcohol dehydrogenases, ATP-dependent helicase HrpB, glutathione *S*-transferases, transcriptional regulators, and ATP binding cassette (ABC) transporter permeases.

Identification of prophages is important for the study of the genome of the MHT1 strain and its genetic potential [30]. In this study, we established that the MHT1 genome harbors two prophage regions (Table S5) with a total size of 55,399 bp using the program Phage_Finder [31]. The lengths of prophage regions 1 and 2 were 25,508 bp (from bp 5,334,617 to 5,360,124, with a GC content of 59.4%) and 29,891 bp (from bp 5,702,675 to 5,732,565, with a GC content of 58.1%), respectively. Sixty-five protein-coding genes were predicted in these two prophage sequences, encoding lysozymes, phage tail proteins, lipoproteins, glycoside hydrolases, and terminases (Table S6).

### Carbohydrate-active enzymes, antibiotic resistance, and substitution rate (*K*_a_/*K*_s_) analysis

Carbohydrate-active enzymes (CAZymes) are proteins that break down carbohydrates into smaller molecules by forming, hydrolyzing, and modifying glycosidic bonds [32]. In this study, we identified 734 genes annotated as CAZyme gene family members in the MHT1 genome (Figure S4). Among them, glycoside hydrolases and glycosyl transferases were the two most abundant protein families, followed by carbohydrate-binding modules, carbohydrate esterases, enzymes with auxiliary activities, and polysaccharide lyases.

We further analyzed antibiotic resistance genes (ARGs) in the MHT1 genome via the Comprehensive Antibiotic Resistance Database (CARD) [33]. We identified 380 ARGs (Table S7); these genes were potentially involved in resistance to fluoroquinolone, polyamine, macrolide, fosfomycin, aminoglycoside, pyrazinamide, cephalosporin, isoniazid, isoniazid, elfamycin, ethionamide, lincosamide, glycopeptide, and rifamycin.

To estimate the evolutionary dynamics of *P. savastanoi*, we employed the MUMmer 3 package [34] to determine orthologous genes between the five strains and calculated the ratio between nonsynonymous mutation rate (*K*_a_) and synonymous mutation rate (*K*_s_) for each gene by the free ratio model [35]. Only four genes showed *K*_a_/*K*_s_ values of > 0.5 (indicative of positive selection); these genes encoded a DUF2244 domain-containing protein, an ATPase, a (2Fe-2S)-binding protein, and a BON (bacterial OsmY and nodulation) domain-containing protein (Table S8).

## Discussion

Although at least 126 whole genomes of *P. savastanoi* strains have been sequenced, assembled, annotated, and uploaded to the NCBI database, none represent a strain that was isolated from an infected kiwifruit plant. *P. savastanoi* strains have been reported to mainly infect woody plants [9–13]. In this study, we demonstrated that kiwifruit, another woody plant, is also a natural host for this pathogen. We isolated *P. savastanoi* strain MHT1 from the leaves of kiwifruit plants that exhibited bacterial canker symptoms, from the Xiache kiwifruit plant base (115°04′N, 24°64′E), Heping County, Heyuan City in Guangdong Province, China. This disease has recently emerged at this location, often showing outbreaks in the spring under rainy and low-temperature weather conditions. So far, from the aspect of disease epidemiology, we still don’t know what is the pathogen transmission vector. However, given changes in local cultivation conditions due to climate change, we do not rule out the possibility that strain MHT1 may an opportunistic pathogenic pathogen of kiwifruit plant.

We sequenced and analyzed the genome of this new strain, providing an essential research basis for studying the characteristics of *P. savastanoi* strains in kiwifruit by allowing an examination of the molecular mechanisms behind the interaction between *P. savastanoi* strain and kiwifruit, with the goal to develop targeted biological control methods. We also selected four additional virulent *P. savastanoi* strains for study. Phylogenetic analysis of MHT1 and these four strains indicated that MHT1 belongs to *P. savastanoi* and exhibits high homology with the genomes of the other four strains. However, the MHT1 strain also differed from the other representative strains by many genes. Notably, two GO terms, ‘antibiotic metabolic process’ and ‘drug metabolic process’, were enriched among genes unique to the MHT1 strain, indicating that it may have strong drug resistance potential. This observation suggests that screening specific bactericidals will be a prerequisite to the effective prevention and control of the diseases caused by MHT1 in production practice. In agreement with this, we identified up to 380 ARGs (Table S7) in the MHT1 genome, further supporting the speculation that the MHT1 strain may exhibit strong resistance to various antibiotics and other chemicals.

In addition, by KEGG pathway enrichment, we determined that the genes unique to the MHT1 strain are mainly involved in the pathways ‘pentose and glucuronate interconversions’, ‘metabolic pathways’, ‘amino sugar and nucleotide sugar metabolism’, ‘nonribosomal peptide structures’, ‘cysteine and methionine metabolism’, and ‘degradation of aromatic compounds’, which may function during infections of kiwifruit and survival in certain environments, such as low temperature and high humidity. It remains to be determined whether the MHT1 strain, like other *P. savastanoi* strains, has a strong ability to infect other woody hosts or whether it can only infect plants in the kiwifruit production areas where it was initially isolated. We noticed the presence of 734 genes in the MHT1 genome that were annotated as CAZyme family genes (Figure S4), among which glycoside hydrolases and glycosyl transferases were the two most abundant families. Whether this feature helps MHT1 adapt to the host plant and environment will require further research to elucidate.

In this study, we investigated conserved type III effectors in *P. savastanoi* MHT1 strains and found that MHT1 presents three effectors that are highly homologous to the RipQ, RipW, and RipBG effectors of *R. solanacearum* strains. This implies that there may be effectors with similar functions in different gram-negative plant pathogenic bacteria and that these effectors may play important roles in the infection of the host, explaining their retention over the course of evolution. The effectors may also have been obtained by horizontal gene transfer, which could provide a molecular basis for MHT1 to acquire stronger pathogenicity or the ability infect more host plants.

## Conclusions

Here, we reported on the complete genome sequence of the *P. savastanoi* strain MHT1, isolated from infected kiwifruit plants showing bacterial canker symptoms. Comparative genomic analysis with *P. savastanoi* strains indicated that the MHT1 genome harbors 230 unique genes enriched in antibiotic metabolism pathways. In addition, 380 and 734 genes in the genome of the MHT1 strain were annotated as antibiotic resistance genes or as encoding carbohydrate-active enzymes, respectively. Furthermore, MHT1 has eight conserved effectors that are highly similar to other effectors from *P. syringae*, *P. amygdali*, and *R. solanacearum* strains. Collectively, these results provide a solid foundation for further studying the host specificity of MHT1 and for screening effective antibiotics for the prevention and control of bacterial canker caused by this pathogen.

## Materials and methods

### Preparation of strains

*Pseudomonas savastanoi* strain MHT1 was isolated from the leaves of one *Actinidia chinensis* plant that exhibited bacterial canker symptoms, on the Xiache kiwifruit plant base (115°04′N, 24°64′E), Heping County, Heyuan City, Guangdong Province of China. Species identification was performed by morphological and molecular analyses. Single colonies were selected after 1 day of culture in LB medium (10 g NaCl,10 g peptone,10 g agar, and 5 g yeast extract, dissolved in 1 L water, pH 7.0) at 28 °C. The selected clones were grown in LB liquid medium for 18 h at 28 °C, followed by centrifugation at 4500 r/min for 5 min to collect cells. Phylogenetic trees were constructed by using software MEGA7.0, according to (NJ) neighbor joining method, the bootstrap replications are 1000.

### Pathogenicity tests

MHT1 cells were cultured in LB medium at 28 °C for 24 h, harvested by centrifugation, and adjusted to 10^8^ CFU/mL in 10 mM MgCl_2_. The suspension was infiltrated into the leaves of *Actinidia chinensis* plants (HongYang kiwifruit and WuZhi kiwifruit) with a needleless syringe. The negative control consisted of infiltration with 1 mM MgCl_2_ without bacteria. For the leaf spraying infection test, the suspension was sprayed onto the abaxial sides of the leaves of *Actinidia chinensis* plants (HongYang kiwifruit). After infection, plants were grown under 100 μmol·m^− 2^·s^− 1^ light irradiance with a 12-h-light/12-h-dark (20 °C/18 °C) cycle in 95% relative humidity. Photographs were taken 8 days post-inoculation. The pathogenic bacteria were isolated and identified from the diseased tissues of HongYang kiwifruit and WuZhi kiwifruit.

### DNA extraction, genome sequencing, and assembly

Genomic DNA from *P. savastanoi* strain MHT1 was extracted with the TIANamp Bacteria DNA Kit (TIANGEN BIOTECH, Beijing, China) and quality-tested on a Qubit 2.0 Fluorometer (Life Technologies, Carlsbad, CA, USA). DNA concentration was measured on a Nanodrop (Thermo Fisher Scientific, Wilmington, USA).

Genome sequencing was performed on a PacBio long-read sequencer (Pacific Biosciences, Menlo Park, CA, USA). SMRTbell libraries were obtained using g-TUBE (Covaris, Woburn, Massachusetts, USA) and end repair. According to the manufacturer’s protocols of the Blue Pippin system (Pacific Biosciences), fragments with sizes larger than 10 kb were selected. The quality and average size of the fragments of the library were estimated using a Qubit 2.0 fluorometer (Life Technologies, Carlsbad, CA, USA) and Bioanalyzer 2100 (Agilent Technologies, Santa Clara, CA, USA). The PacBio Sequel system (Pacific Biosciences, Menlo Park, CA, USA) was used to perform SMRT sequencing. The resulting continuous long reads were used for de novo assembly with the Falcon program (version 0.3.0) [36].

### Functional genome annotation

To predict the open reading frames (ORFs), the NCBI prokaryotic genome annotation pipeline and Prokka (version 1.11) were used [37, 38]. CRISPRfinder (version 4.2.17) was used to estimate CRISPR elements [39]. Ribosomal RNAs (rRNAs), small RNAs (sRNAs), and transfer RNAs (tRNAs) were predicted using the programs rRNAmmer (version 1.2), cmscan (version 1.1.2), and tRNAscan (version 1.3.1), respectively [40–42]. Tandem repeat elements were predicted with the program TRF (version 4.09) [43], interspersed repeat elements were predicted with RepeatMasker (version 4.0.5) [44], and transposons were predicted with TransposonPSI (version 1.0.0) (http://transposonpsi.sourceforge.net/) [45]. Based on sequence similarity, the predicted genes in strain MHT1 were annotated by BLASTN (*E*-value <1e^− 5^), combined with analysis with Gene Ontology (GO), Cluster of Orthologous Groups of proteins (COG), Kyoto Encyclopedia of Genes and Genomes (KEGG), Swissport, and NCBI Non-Redundant Protein (NR) databases. Annotation of protein families was conducted based on Pfam_Scan (version 1.6) and the Pfam database (version 32.0) [46]. Prophages and GIs (gene islands) were predicted using the program Phage_Finder (version 2.0) [28] and Island Viewer (version 4.0) (http://www.pathogenomics.sfu.ca/islandviewer/upload/) [31], respectively. Type III effectors in the MHT1 strain were predicted with the T3E database [20]. The phylogenetic tree was obtained by the REALPHY program (https://realphy.unibas.ch/realphy/) using the whole genome of the MHT1 and other sequenced genomes of *P. savastanoi* strains as input. The Comprehensive Antibiotic Resistance Database (CARD) [33] and the Carbohydrate-Active enZYmes (CAZy) [47] database were used for advanced annotations. The default parameters were used in all analyses.

### Identification of orthologous genes

The alignment of the MHT1 genome and four other representative *P. savastanoi* strains was performed in an all-against-all comparison with the MUMmer 3 package (version 3.3.3) (http://mummer.sourceforge.net/) with default parameters [48]. Orthologous gene clusters in the genomes were estimated with default parameters and with a combination of the programs OrthoMCL (version 2.0) and DIAMOND (parameters of *E*-value <1e^− 5^, query cover > 30%) [49, 50]. The core orthologs and putative proteins in MHT1 were aligned by BLASTP. The score of significant matched proteins of each pair was assigned using a 1 × 10^− 7^ cut-off value [51].

### Substitution rate estimation

The KaKs_Calculator Toolbox software (using the free ratio model with default parameters) (version 2.0) [52] was used to calculate the nonsynonymous mutation rate (*K*_a_) and synonymous mutation rate (*K*_s_). The *K*_a_/*K*_s_ values that were higher than 0.5 [53] were considered genes under positive selection within the *P. savastanoi* strains.

## Supplementary Information

**Additional file 1:** **Table S1**. List of blast with Non-Redundant Protein Database (NR) of strain MHT1. **Table S2**. List of four representative sequenced strains of *P. savastanoi*. **Table S3**. List of identified genes related to type III effectors and the type III secretion system in the MHT1 genome. **Table S4**. List of genes in genomic islands (GIs) of the MHT1 genome. **Table S5**. Identification of prophages in the MHT1 genome. **Table S6**. List of genomic islands (GIs) identified in the MHT1 genome. **Table S7**. Identification of antibiotic resistance genes (ARGs) in the MHT1 genome. **Table S8**. List of genes with a *K*_a_/*K*_s_ values greater than 0.5. **Figure S1**. The Molecular identification of strain MHT1. A and B, the PCR products of the 16S rDNA and *SyrB* gene. M: Marker DNA; 1–5, five duplicate samples. C and D, the PCR products in A and B were retrieved and sequenced. Phylogenetic trees were constructed by using software MEGA7.0, according to (NJ) neighbor joining method, the bootstrap replications are 1000. **Figure S2**. Summary of species distribution of homologs in the Non-Redundant Protein Database (NR). Distribution of different homologous genes, as determined by the NR database. The number of genes involved in the corresponding families is given in parentheses after the species name. **Figure S3.** The hot-map of average nucleotide identity (ANI) between the strain MHT1 and other four strains. Based on the completion of collinearity alignment, pyani [10] was used to calculate the average nucleotide homology of two genome alignment regions between the target genome and the reference related genome. **Figure S4**. Classification of CAZymes by the Carbohydrate-Active enZYmes Database. The horizontal axis represents the function classification of enzymes, and the vertical axis represents the number of encoding genes included in the classification.

## Data Availability

All data generated and analyzed in this study are included in this published article and the supplementary materials. The raw genome sequencing data for *P. savastanoi* strain MHT1 were deposited at GenBank under the BioProject ID: PRJNA736824 (https://www.ncbi.nlm.nih.gov/bioproject/PRJNA736824) and BioSample: SAMN19665368 (https://www.ncbi.nlm.nih.gov/biosample/SAMN19665368/).

## Abbreviations

T3SS: Type III secretion system
T3E: Type III effector protein
NR: Non-Redundant Protein database
GO: Gene Ontology
KEGG: Kyoto Encyclopedia of Genes and Genomes
GI: Genomics island
CAZyme: Carbohydrate-active enzyme
ARG: Antibiotic resistance gene
CARD: Comprehensive Antibiotic Resistance Database
