# Supplementary material for: Complete genome sequence of the kiwifruit bacterial canker pathogen Pseudomonas savastanoi strain MHT1
Source: BMC Microbiol. 2022 Feb 4;22:44. doi: 10.1186/s12866-022-02459-4 (PMC8815115; doi:10.1186/s12866-022-02459-4)
Supplement: Supplementary file 1 — Additional file 1: Table S1. List of blast with Non-Redundant Protein Database (NR) of strain MHT1. Table S2. List of four representative sequenced strains of P. savastanoi. Table S3. List of identified genes related to type III effectors and the type III secretion system in the MHT1 genome. Table S4. List of genes in genomic islands (GIs) of the MHT1 genome. Table S5. Identification of prophages in the MHT1 genome. Table S6. List of genomic islands (GIs) identified in the MHT1 genome. Table S7. Identification of antibiotic resistance genes (ARGs) in the MHT1 genome. Table S8. List of genes with a Ka/Ks values greater than 0.5. Figure S1. The Molecular identification of strain MHT1. A and B, the PCR products of the 16S rDNA and SyrB gene. M: Marker DNA; 1–5, five duplicate samples. C and D, the PCR products in A and B were retrieved and sequenced. Phylogenetic trees were constructed by using software MEGA7.0, according to (NJ) neighbor joining method, the bootstrap replications are 1000. Figure S2. Summary of species distribution of homologs in the Non-Redundant Protein Database (NR). Distribution of different homologous genes, as determined by the NR database. The number of genes involved in the corresponding families is given in parentheses after the species name. Figure S3. The hot-map of average nucleotide identity (ANI) between the strain MHT1 and other four strains. Based on the completion of collinearity alignment, pyani [10] was used to calculate the average nucleotide homology of two genome alignment regions between the target genome and the reference related genome. Figure S4. Classification of CAZymes by the Carbohydrate-Active enZYmes Database. The horizontal axis represents the function classification of enzymes, and the vertical axis represents the number of encoding genes included in the classification. [file 12866_2022_2459_MOESM1_ESM.zip › Table S8.docx]

Table S8. List of genes which with the *Ka/ Ks* value larger than 0.5.

| GeneID | Description |
| --- | --- |
| J4H89_09545 | DUF2244 domain-containing protein |
| J4H89_19370 | ATPase |
| J4H89_21890 | (2Fe-2S)-binding protein |
| J4H89_24345 | BON domain-containing protein |
